# Olfactory Neuroblastoma: Surgical Treatment Experience of 42 Cases

**DOI:** 10.3389/fsurg.2021.799405

**Published:** 2022-02-01

**Authors:** Xiao Cai, Zhouying Peng, Hua Zhang, Ruohao Fan, Yan Fang, Zhihai Xie

**Affiliations:** ^1^Department of Otolaryngology-Head and Neck Surgery, Xiangya Hospital, Central South University, Changsha, China; ^2^Otolaryngology Major Disease Research Key Laboratory of Hunan Province, Changsha, China; ^3^National Clinical Research Center for Geriatric Disorders, Xiangya Hospital, Central South University, Changsha, China; ^4^Anatomy Laboratory of Division of Nose and Cranial Base, Clinical Anatomy Center of Xiangya Hospital, Central South University, Changsha, China

**Keywords:** olfactory neuroblastoma, endoscopic endonasal surgery, skull base surgery, survival rate, prognosis

## Abstract

**Objective:**

Our purpose was to estimate the safety and effectiveness of the endoscopic endonasal approach (EEA) in olfactory neuroblastoma (ONB) and determine whether preservation of the dura and olfactory bulb could be considered in selected patients.

**Methods:**

We retrospectively reviewed patients diagnosed with ONBs between July 2010 and June 2021 at our institution, and collected data on demographic, disease stage, surgical approach, overall survival (OS), disease-free survival (DFS), and postoperative complications.

**Results:**

The study sample included 42 patients (8 treated for recurrence and 34 initial cases), 28 of which were men and 14 were women with a median age of 47.19 years. The mean duration from the beginning of treatment and follow-up time was 8.91 and 51 months, respectively. Among the 42 patients, 32 had unilateral lesions, and the rest had bilateral lesions. Patient symptoms were predominantly nasal, and four patients presented without any symptoms. The modified Kadish staging was A in three patients, B in 14 patients, C in 17 patients, and D in 8 patients. According to the preoperative examinations, five patients had cervical lymph node metastasis, and no patients had distant metastases. EEA was used in 38 patients, cranioendoscopic approach in 3, and open craniofacial approach in 1. The 5-year OS and DFS rates were 89.1 and 79.2%, respectively. The 2-year OS and DFS rates were both 89.1%. The overall surgical complication incidence was 9.52% (one cerebrospinal fluid rhinorrhea, one cervical hematoma, and two epileptic seizures).

**Conclusion:**

The present results support the importance of earlier treatment for advanced ONB and the fact that it is safe and efficacious to treat ONBs *via* EEA. The preservation of the dura can be considered for select patients—specifically those without skull base involvement and who underwent postoperative comprehensive therapy.

## Introduction

Olfactory neuroblastoma is an extremely rare malignant tumor of the nasal cavity that arises from the olfactory neuroepithelium. It accounts for 3–6% of the nasal cavity and nasal sinus malignancies; although, as contemporary histologic techniques are likely to increase detection, it is difficult to determine the true incidence (1). Although olfactory neuroblastoma (ONB) is an uncommon disease, a portion of its characteristics has been identified. Its incidence does not differ significantly according to gender distribution. It affects a wide range of age groups, but most commonly occurs between 50 and 60 years of age. The tumor can involve peripheral parts such as the paranasal sinuses, cribriform plate, and orbits (2). The most common site of metastasis is the cervical lymph nodes (10–33% of patients), with relatively few distant metastases. Kadish et al. developed the most referenced staging system. This system divides tumors into three groups: group A tumors are limited to the nasal cavity, group B tumors involve the nasal cavity and paranasal sinuses, and group C tumors extend beyond the nasal cavity and paranasal sinuses (3). The modification of this staging system by Morita et al. established group D for tumors with regional (neck lymph nodes) or distant metastases (4).

The standard treatment for ONB is a comprehensive therapy that includes surgical resection and postoperative radiotherapy. En-block resection *via* craniofacial approach (CFA) has been the gold standard surgical modality for ONB previously (5). However, the treatment modalities have changed. Based on remarkable progress in technology, endoscopic endonasal approaches (EEAs) have gained acceptance and become an alternative standard for the surgical treatment of ONB (1, 6–9).

Our purpose was to estimate the safety and effectiveness of EEA as an ONB surgical treatment standard. We also strove to determine whether preservation of the dura and olfactory bulb could be considered in select patients without skull base involvement and if outcomes were similar to those who underwent resection of the dura and olfactory bulbs.

## Materials and Methods

### Patient Characteristics

The study included all patients with ONB who underwent surgery between July 2010 and June 2021 at our institution. Each case was diagnosed *via* histopathological examination. Seven patients who had been treated for recurrence were identified among 42 patients with ONB. Each patient underwent a preoperative endoscopy, a sino-nasal CT scan, MRI, and a CT scan or X-ray of the chest. Tumor staging was based on the Kadish staging system, which was initially based on imaging data and then corrected after surgery based on histological data. All patients underwent surgery by the same surgeon.

### Surgical Technique

There are three surgical methods for treating ONB: EEA, CFA, and the cranioendoscopic approach. The first step is tumor resection of the nasal cavity and sinuses. It is vital to identify the attachment of the tumor origin and resection of the sino-nasal component. The lamina papyracea, cribriform plate, fovea ethmoidalis, planum sphenoidale, dura, brain, olfactory bulbs, and tracts were resected depending on the extent of tumor involvement. The skull base is reconstructed in multiple layers, including the fascia lata of the thigh or mucosa flap, when available.

### Statistical Methods

We studied epidemiological data, treatment options, histologic outcomes, postoperative complications, disease-related or other outcomes, and the course of the disease. Descriptive statistics for scaled values and frequencies of study patients within the categories for each of the parameters of interest were enumerated. OS and DFS rates were determined using the Kaplan-Meier method. The statistical significance of differences between the actuarial curves was evaluated using the log-rank test. Follow-up time was defined as the time from the end date of treatment for the original disease to first recurrence, death, or last contact. For all tests, the significance was set at *p* < 0.05. Statistical tests were performed with the assistance of the Statistical Product and Service Solutions (SPSS) Statistics 24 statistical software application (International Business Machines Corporation, USA).

## Results

### Patients

The clinical and demographic data of the patients are summarized in Table 1. The study sample included 8 patients who had been treated for recurrence (19.05%) and 34 initial cases (80.95%). Included in this study were 28 males (66.67%) and 14 females (33.33%). The average age at presentation was 47.19 years (range = 16–79 years). The mean duration from the beginning of treatment and follow-up time was 8.91 months (range = 5 days−72 months) and 51 months (range = 2–127 months), respectively. Among the 42 patients, 32 (76.19%) had only unilateral lesions and the remainder (23.81%) had bilateral lesions. The order of symptom sequence was epistaxis (*n* = 21), nasal obstruction (*n* = 20), hyposmia or anosmia (*n* = 5), headache (*n* = 5), ocular symptoms (*n* = 5), and pain in the nose (*n* = 3). There were four patients without any symptoms. The modified Kadish staging was A in 3 patients (7.14%), B in 14 patients (33.33%), C in 17 patients (40.48%), and D in 8 patients (19.05%). Five patients (11.90%) had cervical lymph node metastasis, while no patients had distant metastasis at presentation according to the preoperative examination.

**Table 1:** The clinical and demographic data of the patients.

	**Number of patients**	**Percentage (%)**
**Sex**		
Males	28	66.67
Females	14	33.33
**Age**		
>45 years	27	64.29
≤ 45 years	15	35.71
Initial cases	34	80.95
Unilateral lesion	32	76.19%
**Symptom**		
Epistaxis	21	
Nasal obstruction	20	
Hyposmia or anosmia	5	
Headache	5	
Ocular symptoms	5	
Pain of nose	3	
No symptom	4	
**Kadish**		
A	3	7.14
B	14	33.33
C	17	40.48
D	8	19.05
NLN^*^metastasis (before the treatment)	5	11.9

* *NLN, neck lymph node*.

### Operative Findings and Additional Treatment

All patients underwent surgery with curative intent. The treatment modalities are shown in Table 2. There were 38 patients who used EEA (90.48%), 3 used the cranioendoscopic approach (7.14%), and 1 used CFA (2.38%). Of the 42 patients, 31 (73.81%) underwent resection including the dura, part of the brain, olfactory bulbs, and tracts (Table 3). We decided the resection range according to the preoperative images (preoperative endoscopy, a skull-base HRCT scan, MRI) and intraoperative observation which could help us to estimate that if there were skull base involvement. Some patients without skull base involvement estimated by the preoperative images and intraoperative observation also underwent resection including the dura, part of the brain, olfactory bulbs, and tracts, for example, the patient No. 27 (Supplementary Figure 1). Postoperative radiotherapy was performed in 27 patients (64.29%), of whom 14 (51.85%) underwent chemotherapy at the same time. Postoperative radiotherapy was not performed in 15 patients (35.71%), but one of them underwent chemotherapy (6.67%). All symptoms were relieved after the surgery.

**Table 2:** Treatment modality.

	**Number of patients**	**Percentage (%)**
**Surgery methods**		
EEA^*^	38	90.48
Cranioendoscopic approach	3	7.14
CFA^**^	1	2.38
**Resection including dura, part of brain, and olfactory bulbs, and tracts**	31	73.81
**Surgery only**	14	33.33
Comprehensive therapy^***^	28	66.67

* *EEA, endoscopic endonasal approach*;

** *CFA, open craniofacial approach*.

*** *Comprehensive therapy, Surgery and radiotherapy or chemotherapy or both*.

**Table 3:** Treatment strategies used for each resection range.

**Group**	A^*^	B^*^
Total	11	31
**Kadish**		
A	1	2
B	7	7
C	1	16
D	2	8
**Comprehensive therapy**	100%	54.84%
**Hymans**		
1–2	3	17
3–4	3	8
**Surgery methods**		
EEA	100%	87.10%

* *A, Resection without dura, part of the brain, and olfactory bulbs, and tracts; B, Resection including dura, part of the brain, and olfactory bulbs, and tracts*.

### Oncological Outcomes

**Figure 1:**
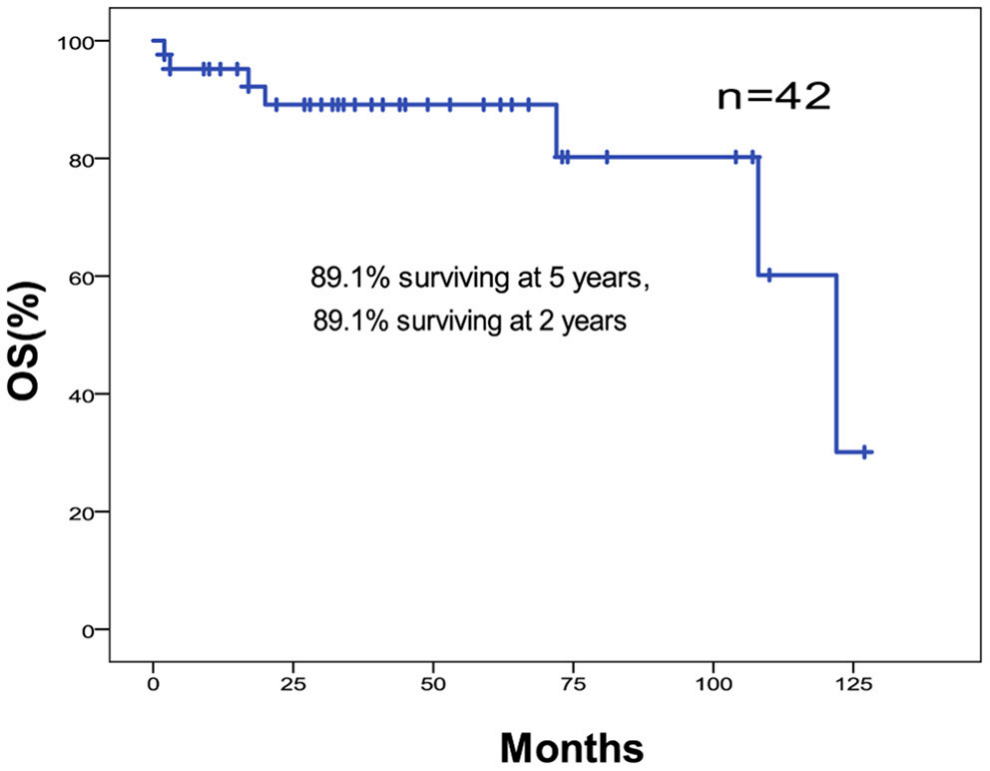
Kaplan-Meier graph of OS of 42 cases of ONB.

**Figure 2:**
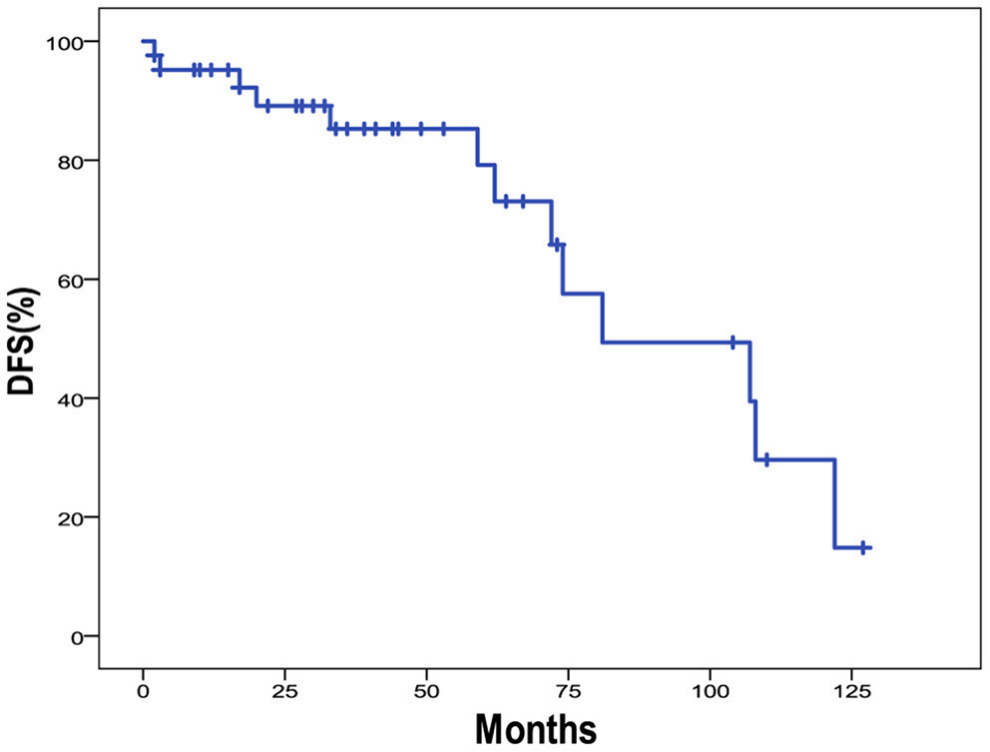
Kaplan-Meier graph of DFS of 42 cases of ONB.

**Figure 3:**
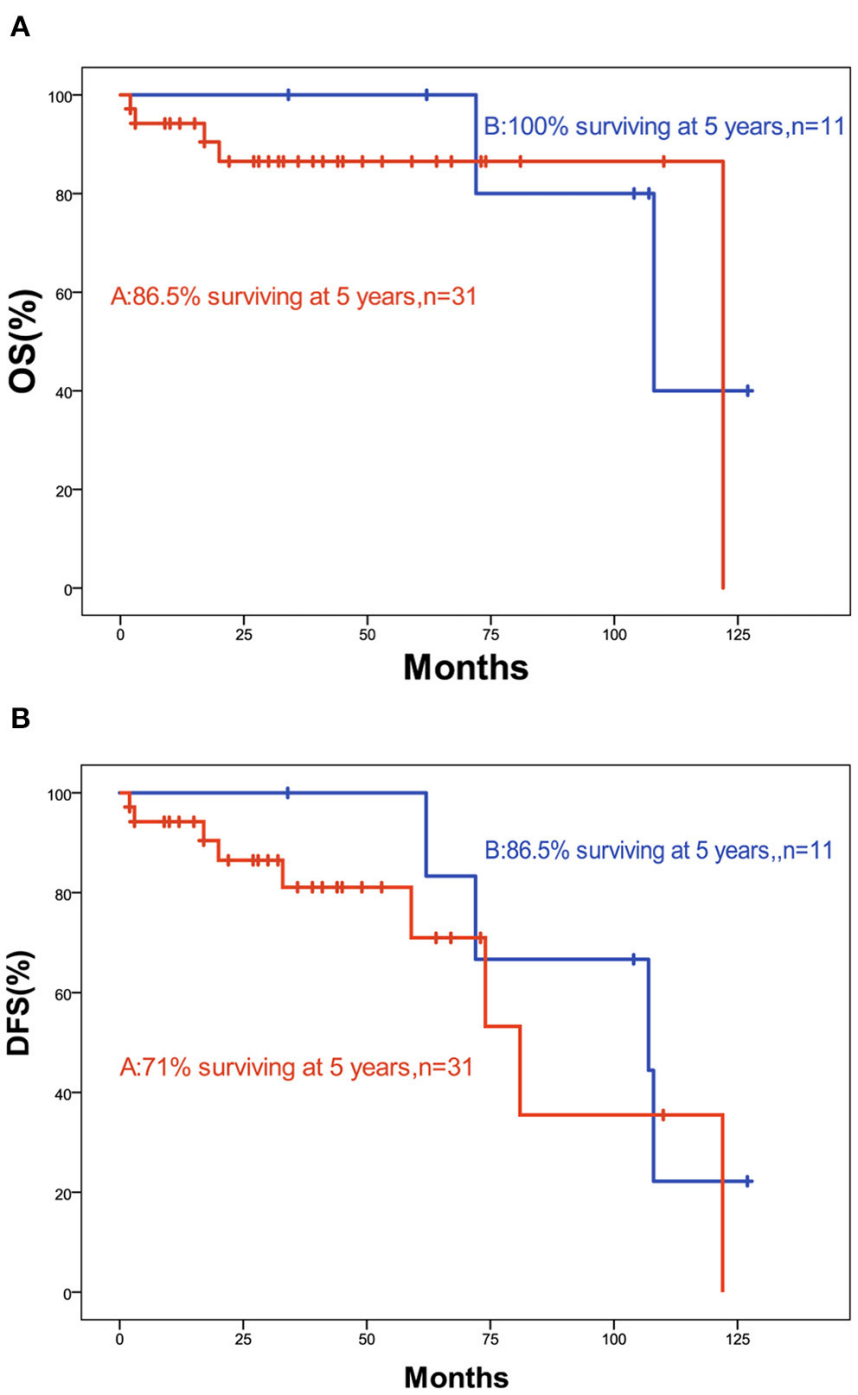
**(A)** Kaplan-Meier graph of OS according to resection; **(B)** Kaplan-Meier graph of DFS according to resection (a, including dura, part of the brain, and olfactory bulbs, and tracts; b, not).

**Figure 4:**
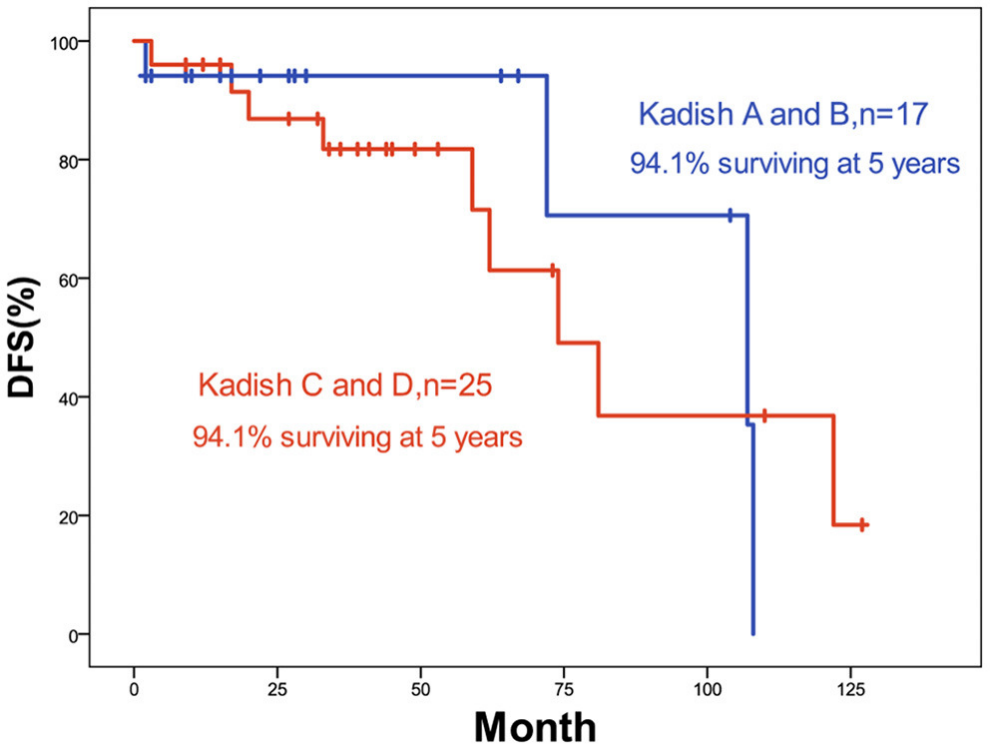
Kaplan-Meier graph of DFS according to Kadish stage.

The 5-year OS and DFS rates were 89.1 and 79.2%, respectively. The 2-year OS and DFS rates were 89.1% (Figures 1, 2). The incidence of local and regional recurrence was 11.9% (5 of 42), and the average recurrence time was 51.6 months (range = 11–102 months). Three patients (7.14%) had cervical lymph node metastasis, while 5 patients (11.9%) had distant metastasis. The 5-year cumulative OS and DFS in patients treated with resection of the dura, part of the brain, and olfactory bulbs, and tracts was 86.5 and 71% compared to 100 and 100% for those who were not by Kaplan Meier (Figure 3). There was no significant difference in DFS between patients who underwent open and endoscopic surgery (*p* = 0.44). The DFS of patients was separately assessed according to the modified Kadish stage of tumors (“A and B” or “C and D”). Although the 5-year DFS of patients with more advanced tumors (71.5%) was lower than that of patients with early-stage tumors (94.1%), the differences were not statistically significant (*p* = 0.7) (Figure 4).

### Complications

The overall surgical complication incidence was 9.52% (4 of 42). One patient had cerebrospinal fluid rhinorrhea, and one had a cervical hematoma. Both patients underwent another surgery. Two patients had epileptic seizures.

### Representative Case

**Figure 5:**
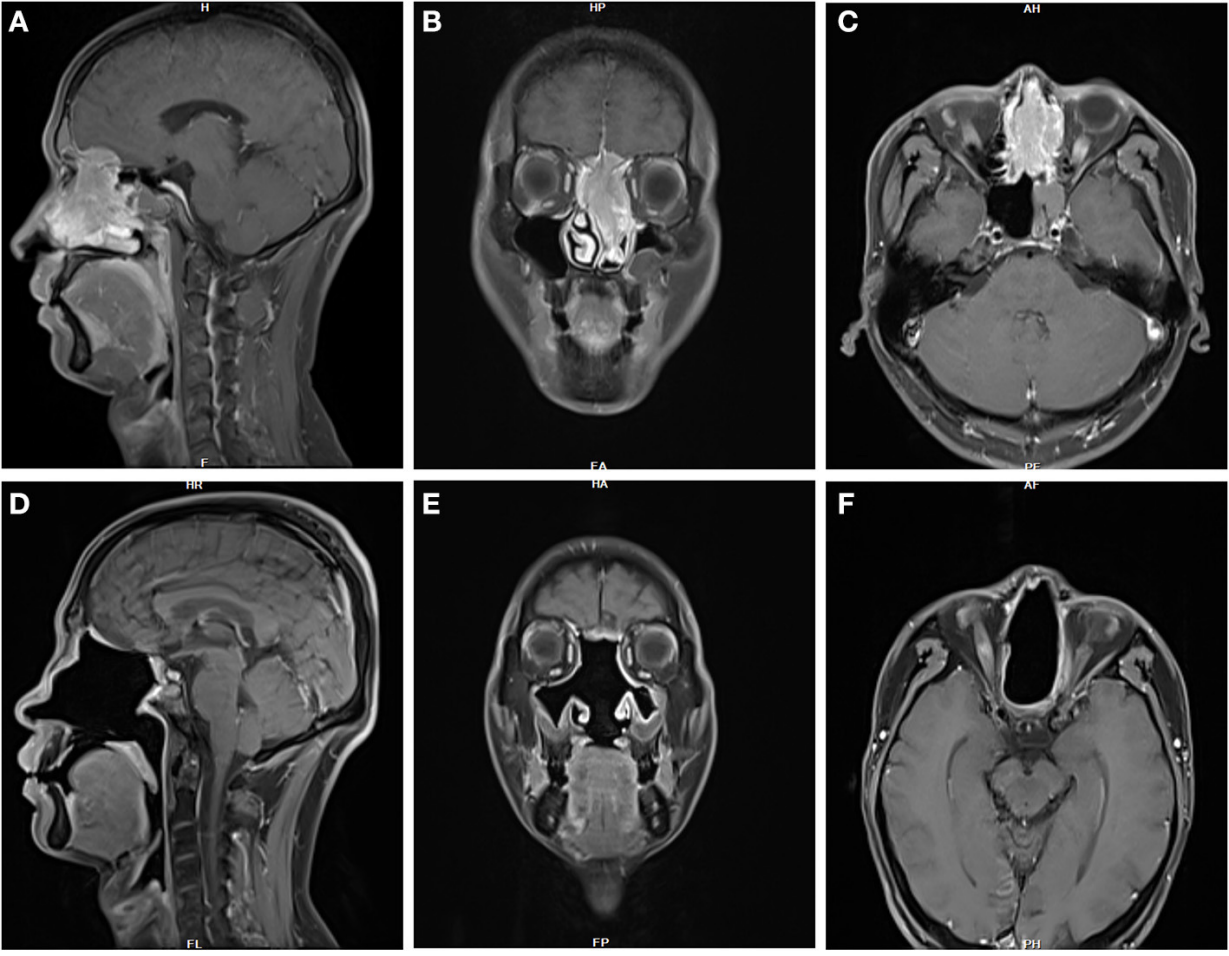
**(A–C)** MRI imaging in this 41-year-old male patient with persistent nasal obstruction revealed a large, left nasal mass. **(D–F)** The patient remained disease-free 29 months postoperatively.

A 41-year-old man with persistent nasal obstruction had a left nasal mass (Figure 5). The patient underwent an EEA for tumor resection. Dural resection was performed, and the brain appeared uninvolved. Radiotherapy and chemotherapy were administered postoperatively. No postoperative complications were observed. The patient remained disease-free 29 months postoperatively.

## Discussion

The Kadish staging system was established in 1976 with three grades (A, B, and C) based on the extent of the primary tumor. This was modified to include a new grade (D) for patients with lymph nodes or distant metastases. However, the prognostic value of the Kadish staging system was not constant. Several studies have found that the early Kadish stages (A or B) have favorable survival rates (2, 10, 11) whereas having not (12, 13). In our study, the 5-year DFS of patients with higher grades (C, D) was lower than that of patients with lower grades (A, B). The differences were not statistically significant. All 3 patients who had neck lymph node metastasis after the therapy had the highest grades (C, D) at first, and only 1 of 5 patients who had distant metastasis after therapy had the lower grade (B) at first. These findings emphasize the importance of earlier treatment of advanced ONB.

The gold standard treatment for sino-nasal tumors since it was first described by Ketcham et al. (14) was open craniofacial resection and radiotherapy. Recently, EEA has been accepted as a common surgical method as an alternative to CFA. Previous reports have indicated that endoscopic resection can replace traditional craniofacial resection in select patients with ONBs (1, 6–9). Rimmer et al. reviewed 95 patients with ONB for a mean follow-up of 89 months, who were treated with endoscopic or craniofacial resection and reported no significant difference in outcomes between endoscopic and craniofacial resection (2). A recent meta-analysis of 609 patients with ONBs concluded that endoscopic resection has a comparable control rate to craniofacial resection (10). In our study, there was no significant difference in the 5-year DFS between endoscopic and craniofacial resection. Functional preservation and fewer complications should be noted as advantages of EEA in comparison to traditional CFA. There was only one patient who had a cervical hematoma and one of two patients in our study, who had epileptic seizures after surgery, underwent CFA. Of the possible post-operative complications, CSF leak is the most relevant and was found in ~10% of cases in previous studies (2, 15–17). In the present study, only one CSF leak was observed, suggesting the safety of our surgical modality. However, long-term observational studies are required to validate this finding, particularly for local recurrence.

Although postoperative radiotherapy is standard, there are some exceptions like the small tumors with good prognosis and extensive surgical resection both visually and histologically (including some ONBs) (18). We advised all the patients to go to the oncology department and ask for comprehensive therapy like radiation therapy. And the oncologists evaluated if the patients need and are able to endure comprehensive therapy according to our surgery and patients' physical condition. In our study, comprehensive therapy was not performed in 14 patients.

Traditionally, resection of the cribriform, dura, part of the brain, olfactory bulbs, and tracts, regardless of tumor stage, has been the standard for all ONB treatments. This is due to two reasons. First, the gold standard treatment for sino-nasal tumors is open craniofacial resection. The development of endoscopic surgery allows for resection of the intranasal tumor and cribriform plate bone while avoiding unnecessary resection of the dura and olfactory bulb, and it is now the treatment for select sino-nasal tumors. Second, the theory that the origin of the tumor is the olfactory neuroepithelium in the superior nasal vault and cribriform is widely held, and many surgeries are considered incomplete unless the dura and olfactory apparatus are resected. The olfactory neuroepithelium is also dispersed throughout the superior turbinate, middle turbinate, nasal cavity, and numerous reports of ectopic origins of ONB have been reported in recent studies (19, 20). Besides the low rates of complications, there was no difference in survival in our study in patients treated with or without resection of the dura and olfactory bulb, suggesting that without skull base involvement, the morbidity of dural resection could have been avoided in selected patients. In our study, we decided the resection range according to the preoperative images (preoperative endoscopy, a sino-nasal CT scan, MRI) and intraoperative observation to estimate the presence of skull base involvement. All patients in whom no resection of the dura and olfactory bulb(s) was performed had no skull base involvement, and they underwent postoperative comprehensive therapy (radiotherapy, chemotherapy, or both).

Long-term observation is critical to determine the oncological outcomes of ONBs. The mean follow-up period in the present study was 51 months, which is not sufficient to evaluate the effect of the intervention on prognosis. Rimmer et al. reported that after an average of 49 months, local and regional recurrence occurred (2) and Nalavenkata et al. reported an average of 60 months (21). Therefore, continuous and careful observation is necessary for our study.

## Conclusion

The present results support the importance of earlier treatment of advanced ONB and the thesis that it is safe and efficacious to treat ONBs *via* EEA. The present results also suggested that preservation of the dura may be considered in select patients without skull base involvement and who were able to undergo postoperative comprehensive therapy. This should be reassessed following long-term observations of oncologic outcomes in our series.

## Data Availability Statement

The original contributions presented in the study are included in the article/Supplementary Materials, further inquiries can be directed to the corresponding author.

## Ethics Statement

The study was reviewed and approved by The Medical Ethics Committee of Xiangya Hospital of Central South University. Ethical review and approval was not required for the study on human participants, in accordance with the local legislation and institutional requirements.

## Author Contributions

ZX, HZ, and RF contributed to conception and design of the study. HZ, RF, and YF organized the database. XC performed the statistical analysis and wrote the first draft of the manuscript. ZP and ZX wrote sections of the manuscript. All authors contributed to manuscript revision, read, and approved the submitted version.

## Conflict of Interest

The authors declare that the research was conducted in the absence of any commercial or financial relationships that could be construed as a potential conflict of interest.

## Publisher's Note

All claims expressed in this article are solely those of the authors and do not necessarily represent those of their affiliated organizations, or those of the publisher, the editors and the reviewers. Any product that may be evaluated in this article, or claim that may be made by its manufacturer, is not guaranteed or endorsed by the publisher.

## Supplementary Material

Supplementary Figure 1 All the pictures were from patient No. 27. **(A)** HES × 5: Pathological picture of the olfactory bulbs. And the olfactory bulbs were not involved. **(B)** HES × 20: Pathological picture of the olfactory bulbs. **(C)** HES × 5: Pathological picture of tumor tissues. **(D)** The tumor cells were positive for Ki-67.
