# Supplementary figures and images for: Olfactory Neuroblastoma: Surgical Treatment Experience of 42 Cases
Source: Front Surg. 2022 Feb 1;8:799405. doi: 10.3389/fsurg.2021.799405 (PMC8845042; doi:10.3389/fsurg.2021.799405)

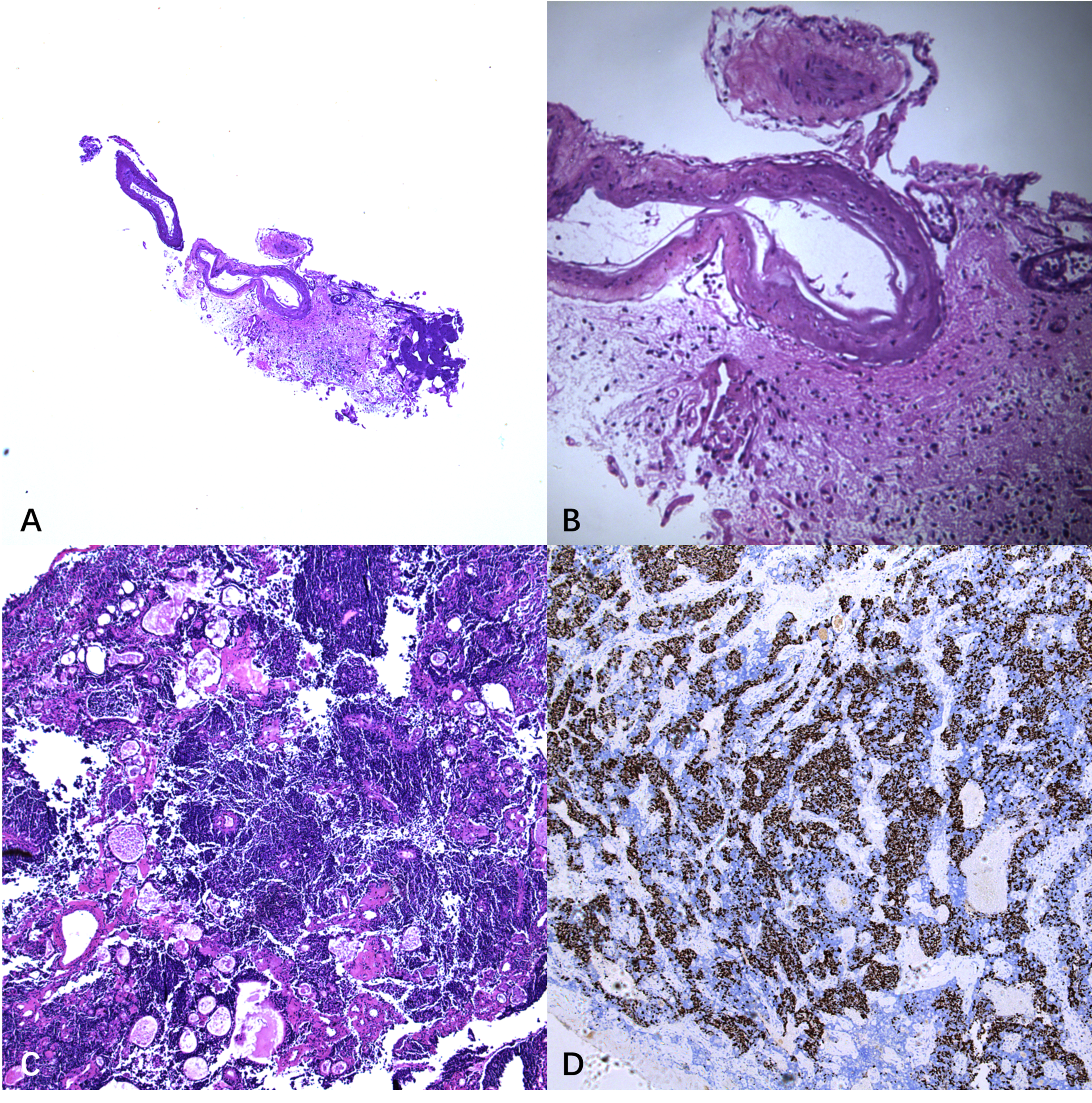

Supplement: Supplementary Figure 1 — All the pictures were from patient No. 27. (A) HES × 5: Pathological picture of the olfactory bulbs. And the olfactory bulbs were not involved. (B) HES × 20: Pathological picture of the olfactory bulbs. (C) HES × 5: Pathological picture of tumor tissues. (D) The tumor cells were positive for Ki-67. [file Image_1.tif]
